# Immunoexpression of the GCDFP-15 Marker in Different Grades of Breast Carcinoma

**DOI:** 10.30699/ijp.2023.558196.2945

**Published:** 2023-03-22

**Authors:** Faeze Shirian, Parvin Kheradmand, Nastaran Ranjbari, Hodjatollah Shahbazian, Seyed Mahmoud Latifi

**Affiliations:** 1 *Department of Pathology, Ahvaz Jundishapur University of Medical Sciences, Ahvaz, Iran*; 2 *Cancer Research Center, Ahvaz Jundishapur University of Medical Sciences, Ahvaz, Iran*; 3 *Department of Clinical Oncology and Radiotherapy, Ahvaz Jundishapur University of Medical Sciences, Ahvaz, Iran*

**Keywords:** Breast cancer, GCDFP-15 marker, Immunohistochemistry

## Abstract

**Background & Objective:**

During the last decade, biological markers of breast cancer have been considered to predict the degree of histology, behavior, and extent of tumor invasion and the possibility of lymph node involvement. The aim of this study was to evaluate the expression of GCDFP-15 in different grades of invasive ductal carcinoma, as the most common type of breast cancer.

**Methods:**

In this retrospective study, paraffin blocks of tumors of 60 breast cancer patients registered in the histopathology laboratory of Imam Khomeini Hospital in Ahvaz between 2019 and 2020 were reviewed. Information on grade, invasion, stage and lymph node involvement was extracted from the pathology reports and immunohistochemical staining for GCDFP-15 was performed. Data were analyzed by SPSS 22.

**Results:**

GCDFP-15 marker expression was observed in 20 out of 60 breast cancer patients (33.3%). GCDFP-15 staining intensity was weak in 7 cases (35%), moderate in 8 cases (40%), and strong in 5 cases (25%). The patient's age and sex showed no significant relationship with the expression of GCDFP-15 and intensity of staining. Expression of the GCDFP-15 marker was correlated significantly with tumor grade, stage, and vascular invasion (*P*<0.05)) and its expression was higher in tumors with a lower grade, less depth of invasion, and no vascular invasion but unrelated to perineural invasion, lymph node involvement, and tumor size. The intensity of staining for GCDFP-15 showed significant relationship with the tumor grade (*P*<0.0001) but unrelated to the other factors.

**Conclusion:**

GCDFP-15 marker may be significantly associated with tumor grade, depth of invasion, and vascular invasion, thus can be used as a prognostic marker.

## Introduction

Breast cancer is among the most commonly diagnosed cancers and a leading contributor to cancer-related deaths, with an estimated number of 2.3 million new cases worldwide according to the GLOBOCAN 2020 estimates (1, 2). The current treatment of breast cancer has been well developed and improved, but the latest data indicate that breast cancer continues to have an extremely high mortality rate among women worldwide (3, 4). Breast cancer is a highly heterogeneous disease caused by interactions between hereditary and environmental risk factors and characterized by a progressive accumulation of genetic and epigenetic alterations (5). Therefore, there is still an urgent need for rapid diagnosis to improve patient outcomes. Over the last decade, biological markers of breast cancer have attracted the attention of many researchers that can be used individually or in combination to predict the degree of histology, tumor behavior and invasiveness (6), and the possibility of lymph node involvement. A prerequisite for a marker to be accepted as a tumor marker is that it must be relatively specific to the tumor, not present in healthy individuals, and easy to measure (7). Because differential diagnosis between primary and metastatic tumors may be challenging in poorly differentiated breast neoplasms, breast cancer markers may be useful in establishing the original site of origin(8). 

Immunohistochemical assessment of this antigen was used as the basis of diagnostic tests to determine the likelihood of disease recurrence hispathologically, invasive breast tumors can be divided into eight main subgroups: invasive ductal carcinoma (80-70%), invasive lobular carcinoma (5-15%), tubular carcinoma (4%), cribriform carcinoma (3-1%), mucinous carcinoma (1%), and micropapillary, medullary and metaplastic carcinoma (9). One of the specific and sensitive markers in breast cancer is prolactin-induced protein or GCDFP-15, which is a 15 kDa protein that is initially detected in cystic fluid from cystic mastopathy. This marker is not expressed in normal ductal or lobular epithelial cells but is expressed in breast apocrine metaplasia. Apart from breast cancer, very few tumors such as prostate cancer and skin appendix cancer express GCDFP-15 (10, 11). It is therefore specifically used to differentiate breast tumors in women and is often used as an immunohistochemical marker to assess the potential origin of metastatic breast cancer at an unknown primary site. 

GCDFP-15 expression is regulated by androgen receptor, however, little is known about its function (12). A recent study on the expression profile of androgen-stimulated GDCFP-15 in cancer cells expressing GCDFP-15 versus GCDFP-15-negative cells reported an up-regulation of proapoptotic and antiproliferative genes associated with GCDFP15. In breast carcinoma, GCDFP-15 is also used as a marker of apocrine differentiation. Apocrine breast cancer is a rare subtype of invasive ductal carcinoma, which is primarily defined by morphological features such as eosinophilic cytoplasm and abundant granules and has frequent androgen receptor expression (13, 14). Recent studies have shown that GCDFP-15 expression is higher in tumors with favorable prognostic features. GCDFP-15 expression is also higher in androgen-positive and apocrine subtypes (15). Few studies have been performed on the expression of GCDFP-15 in different grades of breast cancer, so the aim of the present study was to investigate the immunoexpression of GCDFP-15 in different grades of invasive ductal carcinoma, as the most common subtype of breast cancer, as well as its relationship with clinical features.

## Material and Methods

**Fig. 1:**
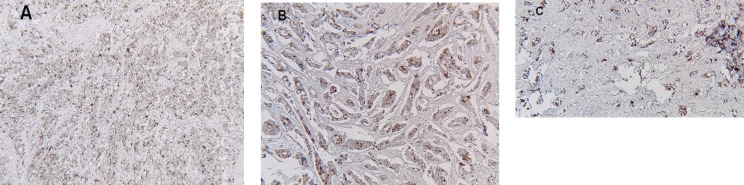
**A.**Strong GCDFP-15 staining in invasive ductal carcinoma **B.** Moderate GCDFP-15 staining in invasive ductal carcinoma **C.** mild GCDFP-15 staining in invasive ductal carcinoma

IHC staining for GCDFP-15 and MAM was performed using polymeric biotin-unfastened horseradish peroxidase (HRP) technique at the Leica Microsystems Bond Max autostainer. In each case, one unstained tissue phase of 4-μm thick that was organized from a consultant paraffin block of a surgically excised TNBC became incubated at 60°C for 20 min. Following heat-triggered epitope retrieval with citrate buffer for 20 min at 100°C, slides have been incubated with mouse monoclonal antibody to GCDFP-15 (clone D6, Covance, Princeton, NJ; 1:100) or MAM (clone 1A5, Biocare Medical, Concord, CA; pre-diluted). The Refine Polymer Detection Kit became used to locate certain antibodies, with 3,3- diaminobenzidine serving because of the chromogen (Leica Microsystems). Slides have been counterstained with Mayer`s hematoxylin. Results have been evaluated concerning fine and poor tissue controls. Any cytoplasmic staining becomes taken into consideration fine. Staining in 5% of tumor cells or more becomes taken into consideration positively. Figures of all types of staining intensity with GCDFP-15 marker, including A: showing strong intensity, B: showing moderate intensity, and C: showing weak intensity (Figure 1). The stains have been reviewed by two pathologists (LH and HZ) independently, and a consensus was reached by re-reviewing the slides when there was any disagreement. 

**Statistical Evaluation**

Statistical analysis was performed using SPSS statistical program 22 (IBM Corporation, Somers, NY, USA). The association between GCDFP-15 expression and clinicopathological factors was analyzed by Fisher’s exact test or Pearson’s Chi-square test. The significance level of the tests was considered less than 0.05. 

## Results

The aim of this study was to evaluate the immunoexpression of GCDFP-15 in different grades of the most common type of breast cancer, invasive ductal carcinoma. Based on the results shown in Table 1, the mean age of patients was 52.80 ± 1.70 years, included 59 (98.3%) females and 1 (1.7%) males. The highest frequency of cancer grades was grade 2,42(70%). The highest depth of invasion was related to PT2 with a frequency of 34 (48.3%). Also, 20 (33.3%) cases showed positivity for GCDFP-15 marker .

**Table 1:** Frequency of distribution of clinicopathological data in the patients with breast cancer

Variable	Level	Number(%)
Age(years)	<50	25 (41/7)
	>=50	35 (58/3)
Gender	Female	59 (98/3)
	Male	1 (1/7)
Grade	1	12 (20)
	2	42 (70)
	3	6 (10)
Depth of invasion	PT1	12 (20)
	PT2	29 (48/3)
	PT3	13 (21/7 )
	PT4	6 (10)
Vascular invasion	-	31 (51/7 )
	+	29 (48/3)
Perineural invasion	-	52 (86/7)
	+	8 (13/3)
Lymph node involvement	-	27 (45)
	+	33 (55)
GCDFP-15 marker expression	negative (staining less than 5% of cells)	40 (66/7)
	Positive (Staining more than 5% of cells)	20 (33/3)
Staining intensity for GCDFP_15 marker	Weak	7(35)
	Moderate	8(40)
	Strong	5(25)

Based on the results shown in Table 2, the immunoexpression of the GCDFP-15 marker demonstrated a significant relationship with tumor grade, invasion depth and vascular invasion (*P*<0.05), and its expression in lower-grade tumors, less invasion depth, and lack of Vascular invasion was greater. 

**Table 2:** Relationship between GCDFP-15 marker immunoexpression and different variables in the patients with breast cancer

Variable	Level	GCDFP-15 marker expressions		P-value
		Negative (Less than 5% staining) Number(%)	Positive (more than 5% staining) Number(%)	
Grade	1	1(2/5)	11(55)	<0.0001*
	2	34(75)	8(40)	
	3	5(12/5)	1(5)	
Depth of invasion	PT1	5(12/5)	7(35)	0.017**
	PT2	18(45)	11(55)	
	PT3	11(27/5)	2(10)	
	PT4	6(15)	0(0)	
Vascular invasion	_	17(42/5)	14(70)	0.04
	+	23(57/7)	6(30)	
Perineural invasion	_	33(82/5)	19(95)	0.179
	+	7(15/5)	1(5)	
Lymph node involvement Variable	_	15(37/5)	12(60)	0.09
	+	25(62/5)	8(40)	

As Table 3 shows, , none of the high-grade tumors including grades 2 and 3, showed strong staining, instead, it shows that with increasing tumor grade, the intensity of staining is decreased. Intensity pf staining for the GCDFP-15 marker was not associated with depth of invasion, perineural invasion, and lymph node involvement.

**Table 3:** Intensity of immunostaining for GCDFP-15 marker with different factors in breast cancer

Variable	Level	Staining intensity		
		Weak Number (%)	Moderate Number (%)	Strong Number (%)
Grade	1	1(14/3)	5(62/5)	5(100)
	2	5(71/4)	3(37/5)	0(0)
	3	1(14/3)	0(0)	0(0)
Depth of invasion	PT1	5(71/4)	1(12/5)	1(20)
	PT2	2(28/6)	6(75)	3(60)
	PT3	0(0)	1(12/5)	1(20)
	PT4	0(0)	0(0)	0(0)
Vascular invasion	_	5(71/4)	5(62/5)	4(80)
	+	2(28/6)	3(37/5)	1(20)
Perineural invasio	_	7(100)	7(87/5)	5(100)
	+	0(0)	1(12/5)	0(0)
Lymph node involvement	_	6(85/7)	3(37/5)	3(60)
	+	1(14/3)	5(62/5)	2(40)

## Discussion

Gross cystic disease fluid protein 15 (GCDFP-15) or Prolactin Induced Protein (PIP) is a 15 kDa protein that was first identified in cystic fluid mastopathy (16, 17). In apocrine metaplasia of the breast, it is not expressed in normal ductal or lobular epithelium (18, 19). GCDFP-15 is expressed only by very few tumors, such as prostate cancer and carcinomas of skin appendages (20). Thus GCDFP-15 is highly specific for breast differentiation in women and is often used as an immunohistochemical marker to assess the potential breast origin of metastatic cancer of unknown primary origin (16). In addition, GCDFP-15 expression has been shown to be associated with decreased cell proliferation and invasion and increased apoptotic pathways in breast cancer patients (21), although some studies have reported conflicting results and a significant relationship between expression of GCDFP-15 and lymph node involvement has been reported (12). Therefore, some have suggested that GCDFP-15 may play an important role in preventing the development and progression of breast cancer. However, there is little and sometimes conflicting evidence in this regard that requires further study (22).

Based on the results obtained from the present study, the expression of the GCDFP-15 marker by the immunohistochemical technique was significantly associated with tumor grade, depth of invasion, and vascular invasion, and its expression was higher in tumors with a lower grade, less invasive depth, and no vascular invasion. Perineural and lymph node involvement were unrelated, possibly due to the small sample size. The results also showed that the intensity of immunostaining for the GCDFP-15 marker has a significant relationship with tumor grade so that 100% of the tumors with strong staining intensity were low grade (grade 1), which indicates with decreasing tumor grade, the intensity of staining increase. However, staining for the GCDFP-15 marker was not associated with depth of invasion, perineural invasion, and lymph node involvement, which is probably due to the small sample size, which warrant further studies with larger sample sizes. The present findings indicate that the GCDFP-15 marker with higher expression in lower-grade tumors, is casociated wih less invasive depth, and no vascular invasion indicating its effectiveness in diagnosis of tumors with good prognosis. However, more studies are needed to evaluate its relationship with patient outcomes, as well as its prognostic role in different types of tumors. Consistent with the present results in the study of Darb-Esfahani* et al.* (2014), higher rates of GCDFP-15 were observed in lower-grade tumors and negative endocrine status and invasion, and it was stated that GCDFP-15 positive tumors have a favorable prognosis compared to negative tumors (16). The examination with the aid of Luo* et al.* (2013) additionally said that GCDFP-15 expression becomes considerably related to a very good analysis of most breast cancers profiles, along with decrease grade, much less differentiation, and no lymph node involvement, and therefore, it can be feasible to diagnose Tumors with a bad analysis aren't sensitive (23). In the study of Fritzsche* et al.* (2007), the GCDFP-15 marker becomes considerably related to the tumor grade, and has inverse relationship with grade of the tumors. In addition, GCDFP-15 negativity was considerably related to a shorter disease-unfastened survival time in univariate and multivariate analysis (the study by Salinas* et al.,* 2012) additionally reported that the immunohistochemical expression of GCDFP-15 in metastatic breast carcinoma becomes related to breast histological grade and with growing tumor grade; much less expression of GCDFP-15 is observed (24). In contrast to the above studies, one study found that increased expression of the GCDFP-15 marker was associated with apocrine morphology and involvement of grade-independent lymph nodes, tumor size, mitotic index, and ER status (12). In Hall* et al.* (1998) study, positive staining for GCDFP-15 was not associated with axillary lymph node involvement but was significantly associated with tumor differentiation and increased with increased tumor differentiation (25) 

Overall, the results of the present study showed the expression of the GCDFP-15 marker in 33.3% of breast tumors and its expression had a significant relationship with the grade, differentiation, and vascular invasion of the tumor, so its expression is higher in lower grade tumors; while more differentiation and no vascular invasion was observed, so GCDFP-15 can be considered as a marker to determine tumors with good prognosis. 

The limitations of the present study include low sample size, retrospective, and cross-sectional nature, as well as incomplete information in the records of patients (in terms of expression of hormone receptors) therefore recommend More studies with larger sample sizes can be designed prospectively and patients can be followed up for several years. PR and HER2, patient survival, response to chemotherapy are also evaluated (16, 25, 26). 

In the present study, which aimed to determine the expression of GCDFP-15 in common grades of breast cancer, the expression of the GCDFP-15 marker (staining of more than 5% of tumor cells in immunohistochemistry) in 20 patients (33.3%) was observed. Staining intensity for the GCDFP-15 marker in immunohistochemistry was weak in 7 patients (35%), moderate in 8 patients (40%), and strong in 5 patients (25%). In some studies, the expression of the GCDFP-15 marker in different types of breast cancer has been evaluated, which has sometimes led to similar results. In the study of Darb-Esfahani* et al.* (2014) the expression of the GCDFP-15 marker was observed in 239 cases of breast tumors (39.7%), which is similar to the values of this study (16). Similar results were reported in the study by El Hag* et al.* (2017), where GCDFP-15 marker expression was observed in 37% of breast tumors (27). In the study of Luo* et al.* (2013), the expression of the GCDFP-15 marker in primary breast cancers (invasive and in situ) was 31.6%, which was 47.2% in 30.6% of invasive tumors and in situ ductal carcinoma (DCIS) (23). In the study of Bhargava* et al.* (2007) on 121 patients with breast carcinomas, GCDFP-15 expression was reported in 28 cases (23.1%) which is slightly less than the present study(28). In the study of Huo* et al.* (2013), a relatively small percentage of GCDFP-15 positive cases were reported in primary triple-negative (TNBC) (14%) and metastatic (21%) breast cancers (29). However, in Lewis* et al.*'s (2011) study, the GCDFP-15 expression rate in ductal breast cancer was 65-71%, in HER2-positive breast carcinoma was 64%, and in TNBC breast carcinoma was 3% (26). In a study by Fritzsche* et al.* (2007) on 165 patients with primary breast carcinoma, GCDFP-15 expression was reported in 70% of tumors (30). In the study of Mazoujian* et al.* (1978), it was observed that 55% of the studied breast carcinoma was positive for GCDFP-15 marker expressions, which were 23% in breast carcinoma without apocrine features and 75% in breast carcinoma with apocrine features 

## Conclusion

In this study, expression of the GCDFP-15 marker in immunohistochemistry was observed in 20 out of 60 patients (33.3%). Staining intensity for the GCDFP-15 marker was weak in 7 patients (35%), moderate in 8 patients (40%), and strong in 5 patients (25%). GCDFP-15 marker expression was significantly associated with tumor grade, depth of invasion, and vascular invasion, and its expression was higher in tumors with a lower grade, less invasive depth, and no vascular invasion, but it had nothing to do with perineural invasion and lymph node involvement. Staining intensity for the GCDFP-15 marker was significantly associated with tumor grade but not with other variables.

## Conflict of Interest

The authors declared no conflict of interest.
